# Endothelial cell–oligodendrocyte interactions in small vessel disease and aging

**DOI:** 10.1042/CS20160618

**Published:** 2017-02-15

**Authors:** Rikesh M. Rajani, Anna Williams

**Affiliations:** MRC Centre for Regenerative Medicine, University of Edinburgh, Edinburgh, EH16 4UU, U.K.

**Keywords:** dementia, endothelial cells, myelin, oligodendrocytes, small vessel disease

## Abstract

Cerebral small vessel disease (SVD) is a prevalent, neurological disease that significantly increases the risk of stroke and dementia. The main pathological changes are vascular, in the form of lipohyalinosis and arteriosclerosis, and in the white matter (WM), in the form of WM lesions. Despite this, it is unclear to what extent the key cell types involved–the endothelial cells (ECs) of the vasculature and the oligodendrocytes of the WM–interact. Here, we describe the work that has so far been carried out suggesting an interaction between ECs and oligodendrocytes in SVD. As these interactions have been studied in more detail in other disease states and in development, we explore these systems and discuss the role these mechanisms may play in SVD.

## Introduction

Cerebral small vessel disease (SVD) is a disease of the small perforating arterioles in the central nervous system (CNS), which causes cognitive impairment, abnormal gait and balance and depression [1–5]. As well as these symptomatic changes, SVD significantly increases an individual's risk of suffering a stroke or dementia. SVD accounts for 25% of all ischaemic strokes [6], and people with SVD have double the risk of suffering a stroke [7]. SVD is the leading cause of vascular dementia [5,8,9], and individuals with SVD are three times as likely to develop vascular dementia [7]. It has also been shown that many patients with Alzheimer's disease also show signs of vascular dementia, suggesting an underlying SVD [5,10], and the outlook for these patients with dual pathology is worse [5].

The prevalence of SVD is greater than many people realize, as SVD can be asymptomatic but still with the same increased risk of suffering a stroke or developing dementia. Studies that have looked for radiological signs of SVD on MRI in the general population have found that approximately 30% of people over the age of 80 have some signs of SVD, even though they may be undiagnosed and asymptomatic [11,12].

Pathologically, SVD is characterized by lacunar infarcts (small subcortical infarcts) [6,13,14], lacunes (fluid filled cavities) [15] and white matter (WM) lesions [16,17] in the parenchyma. In the vasculature, SVD is characterized by small vessel arteriosclerosis and lipohyalinosis (asymmetric thickening of the wall of blood vessel with deposits of hyaline material) [13,18]. On MRI, SVD is seen by small subcortical infarcts, lacunes, WM hyperintensities (WMH), visible perivascular spaces, cerebral microbleeds [19] and, more recently, leakage of intravenously administered gadolinium tracer through the blood–brain barrier (BBB) [20].

Some pathology similar to SVD is seen as part of “normal” aging, which we define here as changes to the brain associated with older age but not associated with disease. Although WM lesions are not considered a part of normal aging, changes to the structure of myelin sheaths with age have been observed in both humans and animal models. Myelin has been shown to be thinner, with shorter internode lengths and with ultrastructural changes resembling decompaction of the myelin sheath in the brains of older humans and primates [21]. All of these changes are likely to lead to a reduced ability of myelin to increase nerve conduction velocity. It has also been shown that vascular function decreases with age, with associated alterations to vascular ultrastructure and reduced vascular reactivity [22,23]. Although these processes

of aging in the WM and vasculature have not been definitively linked, there is evidence to suggest that physical exercise can improve these measures associated with vascular aging, and can ameliorate the signs of WM aging, at least in mice [24]. The fact that the main brain structures involved in both SVD and normal aging are the WM and the vasculature, and the fact that age is the greatest risk factor for SVD, suggest that similar mechanisms may be involved in these processes. Indeed, it has been suggested that SVD is simply an exaggeration of normal aging. However, the existence of monogenic forms of SVD, which appear at much younger ages [25], highlights SVD as a disease. Perhaps the changes associated with “normal” aging may represent the mild end of the spectrum of this disease, at least in some people. For the purpose of this review, we will be focusing on SVD; however, many of these mechanisms are likely to be similar to those in aging.

Although SVD has been extensively described, the origins of the disease and the link between the vascular and parenchymal pathologies of the disease are unknown. This review will explore the work that has been done to study the potential link between the endothelial cells (ECs) of the vasculature and the oligodendrocytes of the WM, and how these interactions may contribute to disease progression. Before we begin, we must first understand the role that oligodendrocytes and ECs play in the normal brain.

## Oligodendrocytes

Oligodendrocytes are the cells that form myelin in the CNS, the fatty sheath surrounding many axons. These cells derived from oligodendrocyte precursor cells (OPCs) that arise from many different regions of the brain during development and are also present throughout the adult brain [26,27]. One of the functions of the myelin sheath is to allow faster transmission of electrical signals through the axon, as the signal can “jump” between the nodes of Ranvier by saltatory conduction rather than propagating through the axon [28,29]. This function is carried out by myelin sheaths forming multiple layers of compacted cell membrane wrapped around the axon [30], increasing the effective resistance of the axon membrane and allowing the electrical signal to travel faster along the axon. The composition of myelin, being predominantly formed of a number of lipids, most notably cholesterol, allows it to carry out this insulating function [31]. Thus, a loss of myelin leads to slower nerve conduction with subsequent functional effects such as motor or cognitive impairment.

More recently, it has been shown that oligodendrocytes also play a role in providing metabolic and trophic support to the axon. Numerous trophic factors secreted by oligodendrocytes have been shown to increase neuronal survival *in vitro* and *in vivo*, including insulin-like growth factor 1 (IGF-1) and brain-derived neurotrophic factor (BDNF) [32,33]. As well as compacted membranes, myelin sheaths have a cytoplasmic channel running through all the layers that comes to rest close to the axonal surface. Through these channels and via lactate transporters, oligodendrocytes provide the axons with lactate, a precursor to pyruvate, from which energy can be derived [34,35]. Transgenic mice engineered to have oligodendrocytes that do not express the monocarboxylate transporter 1 (MCT1), a lactate transporter, show an axonopathy without any change in myelination [34]. Thus, a prolonged lack of metabolic support of axons by oligodendrocytes or absence of oligodendrocytes from axons that were previously myelinated can cause deterioration and death of the axon.

Changes to oligodendrocytes, seen as a loss of WM integrity, are an important aspect of SVD, both histopathologically manifesting as WM lesions and radiologically as WMH. WMH have been shown to correlate strongly with many of the clinical symptoms of SVD, including disrupted gait [1] and cognitive impairment [7]. This suggests that a clearer understanding of what is happening to oligodendrocytes in SVD could be crucial to understanding the clinical aspects of the disease.

## Endothelial cells

ECs are the cells that form all blood vessels in the body. Throughout most of the body, blood vessels are very permeable, allowing perfusion of tissues with required nutrients and solutes and access for the various cells of the immune system to pass in and out of the circulation [36,37]. However, within the brain, ECs are part of a specialized structure known as the BBB. This protects the brain from both invading pathogens and circulating immune cells that may enter and cause damage, and prevents fluctuations in ionic concentrations and signalling molecules in the blood from affecting the normal functioning of the brain [38,39]. Damage to this barrier can therefore cause damage to the cells of the brain, either by allowing the entry of pathogens or immune cells or through toxic changes in solute concentrations. Maintenance of this barrier is important to maintain not only the stable function of the brain but also to protect the limited supply of adult neural stem cells without which the brain's capacity to repair is restricted [40,41].

The primary defence of the BBB is formed by tight junctions (TJs) between ECs. These hold the ECs closer together than adherens junctions present between ECs throughout the body and are in the form of homodimers of TJ proteins of two adjacent EC membranes. In the brain, there are three major families of TJ proteins: occludins, claudins and junctional adhesion molecules [42–44]. Recently, lipolysis-stimulated lipoprotein receptor (LSR, also known as angulin-1) has also been added to this list as a TJ protein that forms a homotrimer at the point where three ECs make contact [45]. Of these TJ proteins, claudin-5 is thought to be the tightest, limiting the pore size of the BBB to <800 Da [46] and found more highly expressed in brain ECs than in other ECs in the body [47]. All of these transmembrane proteins are anchored to the cytoskeleton by intracellular zonula occludens proteins [48].

As well as the TJs between ECs, another layer of the barrier is formed by gap junctions between astrocyte end-feet that surround the small vessels in the brain [49]. Although these gap junctions do not form as tight a junction as TJs, and so do not prevent the passive movement of solutes across the barrier, they provide an extra barrier against cells that have passed through the EC barrier by active processes.

ECs in the brain are highly specialized for this role of forming the BBB. As the BBB prevents cells and molecules from passively crossing into the brain, the ECs of the BBB are adapted to allow for active movement of those that are needed. Thus, they are highly polarized, expressing very different proteins on their luminal and parenchymal surfaces [50,51]. One of these is the GLUT-1 transporter, which transports glucose across the BBB [52]. When leucocytes are required in the brain, receptors to which they can bind such as intercellular adhesion molecule 1 (ICAM-1) are expressed on the luminal surface of ECs [reviewed in 47] and these bound leucocytes can then pass through the EC (transendothelial migration) into the brain [53,54].

ECs also adapt to regulate blood flow to different regions of the brain in response to metabolic need. They do this by releasing nitric oxide (NO), which causes relaxation of the contractile smooth muscle cells and pericytes surrounding the blood vessels. NO is produced by NO synthase (NOS) proteins, of which the dominant form in ECs is endothelial NOS (eNOS). When this process is dysregulated, ECs become dysfunctional. As well as reducing vascular dilation, endothelial dysfunction can also cause smooth muscle cell proliferation on the abluminal side of ECs [55] and platelet adhesion on the luminal side [56], further constricting the vessel. Dysfunctional ECs also secrete factors including vascular endothelial growth factor (VEGF), tumour necrosis factor-α (TNF-α) and endothelin 1 (ET-1) that may have effects on other cells within the brain [57–59]. Finally, endothelial dysfunction can cause leakiness of the BBB, at least in part secondary to EC proliferation [60–63].

Changes to the vasculature have been central to the characterization of SVD, with features such as arteriosclerosis and lipohyalinosis considered as classical pathological signs of SVD [13]. BBB leakiness has previously been shown to be a common feature of SVD [20,64], and though it is not known if this predates WM damage, leakage of cells and blood components from the systemic circulation into the brain parenchyma is unlikely to be beneficial. Evidence has also begun to emerge that endothelial dysfunction may play a role in SVD. Markers of endothelial dysfunction, such as ICAM-1, have been found at elevated levels in the blood of SVD patients [65–68] and genetic polymorphisms in the gene for eNOS, which are associated with reduced eNOS activity, increase the risk of SVD [69,70].

## Endothelial cell–oligodendrocyte interactions in human small vessel disease

Despite the key roles that ECs and oligodendrocytes play in SVD, the interaction between these two brain components has been little studied in this disease. Although one can speculate as to whether vascular changes, such as BBB damage or endothelial dysfunction, which change the chemical balance of the brain may affect OPCs in SVD, the evidence for this is lacking. Part of the reason for this paucity of data is the difficulty in directly studying these components in living humans. This is compounded by the fact that we cannot predict who will go on to develop SVD, and so cannot even try and determine the order of the different pathological changes. Although monogenic forms of the disease do make this possible to some extent, there are not yet any data as to the order of vascular and WM changes in sporadic SVD. There are however a number of animal models of SVD from which greater detail about the mechanisms of these different aspects of SVD can be gleaned.

## Endothelial cell–oligodendrocyte interactions in animal models of small vessel disease

There are a number of different rodent models of SVD, modelling both the sporadic form of the disease and some genetic forms, which have been reviewed extensively elsewhere [71,72]. Though many of these models do not recapitulate the whole disease, there are three models in particular that have been shown to model both the vascular and WM components of the disease, and that may provide some clues to the interactions between them.

The first of these models is the stroke prone spontaneously hypertensive rat (SHRSP). This is an inbred rat, originally derived from the Wistar Kyoto rat, and is considered to be one of the most representative animal models of sporadic SVD [71–73]. These animals spontaneously develop malignant hypertension, between the ages of 6 and 12 weeks [74], classical SVD pathology including lipohyalinosis and WM loss between 8 and 12 weeks of age, and strokes from 20 weeks of age [73,75,76]. It has been shown that these rats have reduced claudin-5, a key TJ of the BBB, at an early age of 5 weeks [73]. This far predates the earliest measured reduction in myelin basic protein (MBP), a key component of the myelin sheath, at 21 weeks [77]. Though this suggests that EC changes predate WM damage, there are a number of aspects of this model that require further study. Firstly, the oligodendrocytes and their precursor cells have not been studied in detail. It is possible that changes in these cells could occur before the apparent deficits in MBP and possibly even before the breakdown of the BBB. Secondly, studies in this model have only given a descriptive account of the pathological changes with some hints of their order. There has been little study of the mechanisms that might link these changes, and give some clue for the importance of EC–oligodendrocyte interactions in the disease.

Another model of sporadic SVD that displays both vascular and WM phenotypes is the inducible mouse model of chronic cerebral hypoperfusion. This is induced by placing microcoils on the common carotid artery causing chronic constriction of this blood vessel. This model shows many features of SVD, including lacunar infarcts and WMH on MRI, myelin damage and vascular pathology in the form of lipohyalinosis and BBB damage [78,79]. The inducible nature of this model makes it easier to study mechanisms and timings of subsequent cerebral damage. The fact that alterations to the vasculature produce myelin damage resembling that seen in SVD in this model demonstrates that blood vessel changes may be important for the formation of WMH in human SVD. In this model, disruption of the paranodes, where the myelin sheath contacts the axon through paranodal loops, far predates leakiness of the BBB and suggests that WM pathology is not caused by toxic leakage of blood components through the BBB. As this model is induced by chronic blood vessel constriction and hypoperfusion, it is believed that these changes are caused by a reduction in oxygen and nutrients reaching the WM. However, direct signalling between ECs and cells of the WM has not yet been studied in this model.

The models described above hint at an interaction between ECs and WM in SVD, and other models also at least suggest that blood vessel changes and WM pathology are linked. The common form of inherited SVD is cerebral autosomal dominant arteriopathy with subcortical infarcts and leukoencephalopathy (CADASIL), which has a well-characterized mouse model. CADASIL is caused by a mutation in the *NOTCH3* gene [80], which leads to an accumulation of the extracellular domain of NOTCH3 (NOTCH3^ECD^) in the basement membrane around cerebral blood vessels [81]. The animal model of this disease shows much of the WM and vascular pathology seen in the human disease, with vascular dysfunction predating WM lesions [81]. In the mouse model, it has been shown that the plaques of NOTCH3^ECD^ around the blood vessel lead to an accumulation of tissue inhibitor of metalloproteinase 3 (TIMP3) and vitronectin (VTN) [82]. By crossing the CADASIL mouse with knockout mice for either *Timp3* or *Vtn*, it has been shown that removal of TIMP3 rescues the vascular pathology, but not the WM pathology, whereas removing VTN rescues the WM pathology but not the vascular pathology [83]. However, the cellular basis of these molecular changes is unclear. According to one RNA-Seq database [84], NOTCH3, TIMP3 and VTN are normally expressed by ECs at the RNA level and the increased expression of TIMP3 and VTN proteins, which occurs in CADASIL, co-localizes with NOTCH3^ECD^ aggregates around blood vessels, a pathological marker of the disease. As these two pathways have independent effects on WM and vascular pathology, it is unclear whether there is a direct interaction between ECs and oligodendrocytes. However, it is conceivable that EC overproduction of at least VTN may influence oligodendroglia, directly or indirectly. However, the details of the mechanism of this interaction are unknown.

Evidence from other monogenic forms of SVD also hints at an interaction between ECs and oligodendrocytes. *COL4*-related angiopathies are caused by mutations in the *COL4A1* or *COL4A2* genes, which cause defects in the α 1 or α 2 chain of collagen IV respectively. Collagen IV is a key component of the basement membrane that supports blood vessels [85]. Both humans and mouse models with mutations in these genes show pathology similar to SVD, including vascular and WM changes [86,87]. The high expression of both *COL4A1* and *COL4A2* by brain ECs [84] suggests that disrupted signalling from the ECs may be involved in the WM changes seen in this form of the disease.

Another monogenic form of SVD is cerebral autosomal recessive arteriopathy with subcortical infarcts and leukoencephalopathy (CARASIL), caused by a loss of function mutation in the gene HtrA serine peptidase 1 (*HTRA1*) [88]. Patients with these mutations show SVD pathology, including vascular alterations, subcortical infarcts, WM changes and cognitive defects [25,89,90]. Although an animal model of this form of SVD has yet to be developed, it has been shown *in vitro* that HTRA1 is involved in regulating the levels of transforming growth factor β (TGF-β) [91,92]. TGF-β has been shown to play a role in both endothelial dysfunction [93] and BBB integrity [94]. Thus, a misregulation of TGF-β due to a loss of HTRA1 in CARASIL may lead to altered signalling from the ECs to other cell types, including oligodendrocytes.

## Endothelial cell–oligodendrocyte interactions in other models

Although studies of EC–oligodendrocyte interactions in SVD and its models may be limited, there has been more extensive investigation of this in other systems. One of the first studies to show interaction between ECs and OPCs was by Arai and Lo [95]. This study coined the term oligovascular niche, as an equivalent of the neurovascular niche that had been previously described [96,97], and showed that ECs play a role in the support of OPCs. By adding EC conditioned media to OPCs, they demonstrated that ECs secrete factors that promote OPC proliferation in normal conditions. Furthermore, using oxygen-glucose deprivation (OGD) to model OPCs under disease conditions, they showed that factors secreted by ECs can encourage OPC survival. This study suggested that fibroblast growth factor (FGF) and BDNF may be involved in mediating these effects through the Akt pathway. A recent study suggests that these growth factors may be contained in extracellular vesicles, which have been shown to be released by ECs and taken up by OPCs, playing an important role in increasing OPC survival, proliferation and motility [98]. More importantly, these data demonstrate that a diseased vasculature could alter the survival of oligodendroglia, potentially providing a link with WM changes relevant in SVD.

This interaction between ECs and OPCs does not occur only in one direction. Using animal models of neurofibromatosis, with deleted *Nf1* or constitutively active Ras present specifically in mature oligodendrocytes, it has been demonstrated that oligodendrocyte changes can also affect ECs [99]. In this disease model, as well as the expected disruption to myelin, they also found enlarged perivascular spaces and BBB leakage with altered TJs. Mechanistically, they suggested that this was due to an increase in production of reactive oxygen species (ROS) in the oligodendrocytes. The fact that these mutations were oligodendrocyte specific demonstrates clearly that the cause of the dysfunction of the BBB was secondary to the altered oligodendrocytes signalling to the ECs. Other studies have demonstrated the importance of signalling from OPCs to ECs in BBB maintenance using conditioned media *in vitro*, showing that TGF-β1 released by OPCs supports BBB integrity [100], whereas matrix metalloproteinase 9 (MMP-9) secreted by stressed OPCs disrupts the BBB and encourages angiogenesis [101,102].

More recently, further evidence of this bidirectional interaction between ECs and oligodendroglia has been shown during development. Signalling from ECs to OPCs plays a crucial role in OPC localization, as OPCs crawl along or jump between blood vessels to migrate throughout the brain away from the sites where they are formed before differentiating into myelin-forming oligodendrocytes [103]. Disruption of vascular patterning, or disruption of the interaction between Cxcl12 on the ECs and Cxcr4 on OPCs, leads to reduced OPC migration and aberrant localization throughout the mouse brain and spinal cord [103].

It has also been shown that during development signalling from OPCs to ECs plays an important role in angiogenesis [104]. Hypoxic OPCs secrete Wnt7a and Wnt7b. These secreted Wnts act both cell autonomously, preventing OPC maturation and thus maintaining them in the progenitor state, but also on ECs, where they increase *Lef1* expression, EC proliferation and angiogenesis. These two signalling pathways may play complementary roles, where OPC release of Wnts encourages angiogenesis both to provide increased oxygen and nutrient supply then allowing for OPC maturation, and also to provide a migrational pathway for OPCs to move to other areas of the CNS. It is not yet known whether a similar mechanism allows movement of OPCs in diseased CNS tissue, but increased angiogenesis occurs early after demyelination is induced in mouse models [105,106], and neural precursors stimulated to be released from the subventricular zone (SVZ) are highly associated with these vessels en route to the area of damage in the corpus callosum [105]. Increased proliferation of ECs and increased blood vessel density is also seen in WM demyelinated lesions in post-mortem tissue from people with multiple sclerosis (MS) [107,108]. The role of this in either the demyelination of WM or its recovery in MS is unknown, but in a mouse model of demyelination suppression of angiogenesis with anti-VEGF antibodies reduced progenitor cell migration to the demyelinated lesion [105].

## Indirect endothelial cell–oligodendrocyte interactions

As well as interacting directly, it is also possible that ECs and oligodendrocytes may interact indirectly through other vascular-associated cell types. Throughout the brain, capillaries are directly apposed by pericytes, contractile cells from the same lineage as smooth muscle cells present around larger vessels [109]. These cells provide support to the ECs and are vital for the formation and maintenance of the BBB [110,111]. More recently, it has also been shown that pericytes also interact with OPCs [112]. As well as demonstrating that these two cell types are in close contact, it was also shown using conditioned media experiments that pericytes secrete factors promoting OPC survival, and OPCs secrete factors that promote pericyte survival [112]. Thus, OPCs may encourage the maintenance of BBB integrity through promotion of pericyte survival. Similarly, ECs may promote OPC survival through the actions of pericytes. However, pericytes cannot mediate all of the interactions between EC and OPCs, as mice that are genetically modified to lack pericytes still show OPC migration along blood vessels [103].

**Figure 1:**
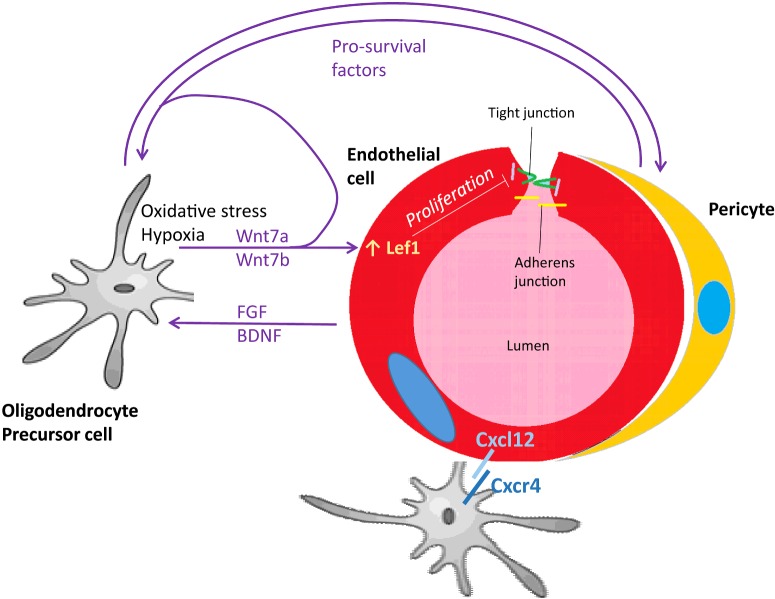
Diagram showing the different mechanisms by which ECs and oligodendrocytes have been shown to interact OPCs that are hypoxic secrete Wnt7a and Wnt7b, which act cell autonomously to prevent OPC differentiation, and on the EC, where they up-regulate the expression of *Lef1*, increasing EC proliferation and angiogenesis [104]. As EC proliferation leads to a leakier BBB [115,116], a similar mechanism could explain how OPCs undergoing oxidative stress cause BBB leakiness [99]. ECs also signal to OPCs, both through secreted factors including FGF and BDNF that increase OPC survival [95], and through cell contact interactions through Cxcl12 and Cxcr4 that allow OPC attachment for migrating along blood vessels [103]. Pericytes, which appose and support ECs, also demonstrate bidirectional signalling with OPCs through secreted pro-survival factors [112].

ECs of the BBB are also surrounded by astrocyte end-feet. As well as providing a second line of defence to the BBB, this proximity also allows for interaction between ECs and astrocytes. Astrocytes are thought to play an important role in inducing the BBB phenotype of brain ECs [38], and regulating vascular responses to neuronal activity [113]. Astrocytes have also been shown to provide functional support to OPCs and secrete factors that protect OPCs during insults including OGD and oxidative stress [114]. Thus, astrocytes have the potential to mediate changes to both ECs and OPCs. Figure 1 summarises current knowledge of these different mechanisms of interaction between ECs and oligodendrocytes.

## Possible mechanisms of endothelial cell–oligodendrocyte interactions in small vessel disease

Although these interactions detailed above have been shown in development and other disease states, they could provide clues as to how EC–OPC interactions may be important in SVD and aging. If we first consider signalling from the oligodendrocytes to the ECs, the data from the oligodendrocyte-specific *Nf1* mutant [99] tie in well with the data from the chronic cerebral hypoperfusion model of SVD [78,79]. Here, oligodendrocyte damage, either from genetic causes or mechanical causes that induce hypoxia, may then alter oligodendroglial signalling, involving Wnt secretion, and lead to increased angiogenesis [104], which may subsequently be the cause of BBB leakage [115,116].

Data from these systems also provide evidence that EC to oligodendrocyte signalling may be important in SVD. As ECs normally release factors that encourage OPC survival and proliferation [95], altered signalling from ECs, through genetic or environmental changes, may make oligodendrocytes more vulnerable to damage, either by reducing their exposure to pro-survival factors or by reducing the ability of the WM to repair after injury. This links with the evidence from humans with SVD that ECs are dysfunctional [65–68] and reduced eNOS activity may even play a causative role [69,70]. Endothelial dysfunction significantly alters the factors that ECs are secreting [57–59], which may affect oligodendrocyte survival.

It is also possible that rather than causing the WM damage in SVD, EC–oligodendrocyte interactions simply exacerbate it. Direct signalling from dysfunctional ECs to oligodendrocytes may alter their ability to withstand or respond to damage from other causes such as hypoperfusion. In other diseases of the WM, such as MS, which have been more extensively studied, OPCs respond to WM damage by proliferating and migrating into the demyelinated lesion before differentiating into remyelinating oligodendrocytes. EC–OPC interactions have been shown to be important for two of these three processes. Firstly, secreted factors from ECs encourage OPC proliferation [95]. Thus, altered EC signalling in SVD may reduce this. Secondly, blood vessels provide an important scaffold along which OPCs migrate during development [103], and it may be that similar mechanisms are used for OPC migration after pathology during adulthood. Altered EC expression of surface proteins or angiogenesis in SVD may impair this migration and reduce the repair process in response to damage.

## Future perspectives

Given the important role that OPCs play in regulating EC function, and that ECs play in supporting OPCs, it is likely that signalling in both directions plays a role in SVD. It is even possible that these bidirectional signals compound each other, exacerbating any initial damage. However, to truly understand the importance of EC–oligodendrocyte interactions in SVD, and to better appreciate how this may influence treatment of the disease, further investigation needs to be carried out in some key areas.

Firstly, a clearer understanding needs to be developed as to the order of the changes in SVD. Although the unpredictability of the disease makes this problematic in people with sporadic SVD, the occurrence of monogenic forms of the disease presents opportunities in this area. As individuals known to have causative disease mutations may be regularly monitored for signs of the disease, a study detailing the order in which radiological signs of the disease appear could provide valuable data.

To determine the order of changes in sporadic SVD, the SHRSP model, detailed above, could be used. As these rats spontaneously and consistently develop SVD, they could be studied in detail from a young age to determine if oligodendrocyte abnormalities appear before the observed reduction in MBP, and how this relates to changes in the ECs.

Secondly, the molecular signalling pathways between ECs and oligodendrocytes, which are specific to SVD, need to be identified. The studies in other models described here provide methods by which these can be clarified either *in vitro* [95,104], using cells isolated from SHRSPs or CADASIL mice or *in vivo* [99,103,104], using SHRSPs, CADASIL mice or inducible SVD models. Although this level of molecular dissection is not possible in humans, any molecules that appear important from *in vitro* and *in vivo* studies can then be studied in post-mortem brains from people with and without SVD.

Finally, it must be understood if either disrupting or altering these signalling pathways can affect the progress or development of SVD. Data from these studies would give us a clear understanding of the importance of EC–oligodendrocyte interactions in SVD, and may lead to the development of successful therapeutic strategies for patients.

## Abbreviations

BBB: blood–brain barrier
BDNF: brain-derived neurotrophic factor
CADASIL: cerebral autosomal dominant arteriopathy with subcortical infarcts and leukoencephalopathy
CARASIL: cerebral autosomal recessive arteriopathy with subcortical infarcts and leukoencephalopathy
CNS: central nervous system
EC: endothelial cell
eNOS: endothelial nitric oxide synthase
ET-1: endothelin 1
FGF: fibroblast growth factor
HTRA1: HtrA serine peptidase 1
ICAM-1: intercellular adhesion molecule 1
IGF-1: insulin-like growth factor 1
MBP: myelin basic protein
MCT1: monocarboxylate transporter 1
MMP-9: matrix metalloproteinase 9
MS: multiple sclerosis
NO: nitric oxide
NOS: NO synthase
OGD: oxygen-glucose deprivation
OPC: oligodendrocyte precursor cell
SHRSP: stroke prone spontaneously hypertensive rat
SVD: small vessel disease
SVZ: subventricular zone
TGF-β: transforming growth factor β
TIMP3: tissue inhibitor of metalloproteinase 3
TJ: tight junction
TNF-α: tumour necrosis factor-α
VEGF: vascular endothelial growth factor
VTN: vitronectin
WM: white matter
WMH: WM hyperintensities
